# Therapeutic Exercise Effects on Activity, Participation and Quality of Life in Individuals With Temporomandibular Disorders: A Systematic Review

**DOI:** 10.1111/joor.14042

**Published:** 2025-06-09

**Authors:** Sarah Scrase, Roma Forbes, Adrienne Lindop, Adrienne Parcher, Louise Rainbird, Alana Dinsdale

**Affiliations:** ^1^ School of Health and Rehabilitation Sciences The University of Queensland St Lucia Queensland Australia

**Keywords:** activity, oral rehabilitation, participation, QOL, temporomandibular disorders, therapeutic exercise

## Abstract

**Background:**

Temporomandibular disorders (TMDs) are common musculoskeletal jaw conditions that can significantly impact individuals' activity, participation and quality of life (QOL). Current clinical recommendations for TMD management encompass therapeutic exercise, although it is unclear how exercise impacts recovery beyond improving individuals' jaw pain and range of motion.

**Objective:**

To investigate the effects of therapeutic exercise on patient‐reported measures of activity, participation and corresponding QOL in individuals with TMD.

**Methods:**

An electronic database search of PubMed, Embase, CINAHL and Cochrane Central was performed on 7 March 2024. Inclusion criteria were peer‐reviewed journal articles written in English that investigated the effect of therapeutic exercise on activity, participation or QOL in individuals with TMD. Risk of bias was assessed, and data were grouped according to outcomes then analysed using a narrative synthesis approach. The confidence in cumulative evidence for each outcome was determined using a modified GRADE approach.

**Results:**

Twelve studies were eligible for this review, comprising a total of 775 participants (mean age = 32.5 years, 79% female). Therapeutic exercise interventions included both global (e.g., aerobic, core, relaxation, postural) and local jaw‐specific (e.g., mobility resistance) exercises. Findings suggest that both jaw‐specific and global exercises may improve activity, participation and QOL in patients with TMDs. These findings should be considered with caution as confidence in cumulative evidence was very low.

**Conclusion:**

Therapeutic exercise may be effective in improving activity, participation and QOL in individuals with TMDs, although further research is needed to improve the quality of the evidence and to direct clinical guidelines.

## Graphical abstract

Graphical representation of review aims, methods and findings.

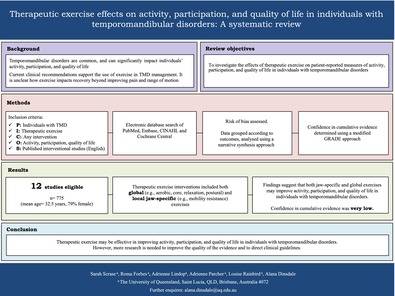

## 1 Introduction

Temporomandibular disorders (TMD) comprise conditions affecting the temporomandibular joint and surrounding structures. They are characterised by symptoms such as orofacial pain, jaw clicking, locking and headaches [1]. TMD can severely limit jaw movement, affecting speaking, chewing and eating, thereby impacting overall health, social participation and psychological well‐being [1]. While up to 70% of the global population may experience TMD symptoms, primarily between ages 20 and 40, most cases resolve spontaneously [2]. However, reports suggest that approximately 12% of individuals with TMD experience symptoms that may require professional treatment [2].

The International Classification of Functioning, Disability and Health (ICF) provides a framework for measuring health and disability experienced by individuals with health conditions, such as TMD [3, 4]. The ICF conceptualises disability as the dynamic interaction between issues in the domains of body functions and structures, activity, participation and contextual factors. In the ICF, ‘body functions and structures’ pertain to the anatomical parts and physiological functions of body systems while ‘activity’ refers to the execution of tasks and ‘participation’ encompasses engagement in life situations. Ultimately, the interplay of these domains is pivotal in determining an individual's overall quality of life (QOL) [3].

Evidence‐based recommendations for TMD management support multi‐disciplinary and multimodal approaches to care that often focus on improving body functions and structures, activity, participation and overall QOL. Management may involve various health professionals, such as dentists, doctors, psychologists and physiotherapists who offer a myriad of interventions including oral splints, medication, cognitive therapies, therapeutic exercise and manual therapy. Current best practice recommendations for musculoskeletal conditions state that exercise should be a consistent feature in management, while other modalities (e.g., manual therapy) should be applied as an adjunct [5]. Clinical guidelines for the management of chronic TMD‐related pain support this, strongly recommending the use of therapeutic exercise in care [6].

Therapeutic exercise is defined by the American Physical Therapy Association (APTA) as the “systematic performance or execution of planned physical movements or activities intended to remediate or prevent impairments of body functions and structures, enhance activities and participation, reduce risk, optimise overall health, and enhance fitness and wellbeing” [7]. Thus, therapeutic exercise includes a range of approaches that are used by health professionals such as stretching, strengthening, motor control and relaxation training. Current evidence suggests that therapeutic exercise improves clinical measures of range of motion (ROM) and pain in individuals with TMD, which align with the body functions and structures domains of the ICF [8]. However, it is unclear whether therapeutic exercise affects the ICF domains of activity, participation, and more broadly, QOL. This exploration is essential for developing more effective and meaningful management strategies for TMD.

This systematic review therefore aimed to investigate the effects of therapeutic exercise on jaw‐related disability in individuals with TMD. The primary focus was on patient‐reported measures of activity, participation and corresponding QOL to address gaps in the literature. The secondary focus was on measures of body functions and structures due to their known interaction with the activity and participation domains [3, 9, 10].

## 2 Methods

### 2.1 Study Design and Registration

This systematic review was conducted in accordance with Preferred Reporting Items for Systematic Reviews and Meta‐analyses (PRISMA) guidelines [11]. The protocol was registered with the Prospective Register of Systematic Reviews (PROSPERO) [CRD42024527414].

### 2.2 Eligibility Criteria

#### 2.2.1 Population

The population of interest was individuals with TMD. Studies including participants of any age diagnosed with TMD using the Diagnostic Criteria for Temporomandibular Disorders (DC/TMD), Research Diagnostic Criteria for Temporomandibular Disorders (RDC/TMD), clinical examination, or self‐reported criteria (e.g., via standardised screening tools such as the 3Q/TMD, or through presence of TMD symptoms such as jaw pain, joint sounds or locking) were eligible for inclusion. Studies were excluded if participants had jaw symptoms associated with other medical conditions (e.g., trigeminal neuralgia, fibromyalgia, cancer, rheumatoid arthritis). Studies involving animals were excluded.

#### 2.2.2 Intervention

The intervention targeted in this review was therapeutic exercise. Studies with any form of therapeutic exercise intervention consistent with the APTA definition described earlier were included, provided that the effects of the exercise could be isolated [7]. These included global and local resistance training, muscle strengthening, stretching, relaxation, motor control and postural control. Studies were excluded if exercise was part of a multimodal intervention where data provided did not permit independent analysis of the effect of the exercise component.

#### 2.2.3 Comparator

All comparators were considered, including standard care, no care and alternative interventions.

#### 2.2.4 Outcome

The primary outcomes of interest were patient‐reported measures of activity, participation and QOL. In studies reporting at least one primary outcome, secondary outcome data were also considered. Secondary outcomes included clinical measures of the functions and structures of the jaw, such as jaw pain and ROM.

#### 2.2.5 Study Type

This systematic review included randomised controlled trials (RCTs), non‐randomised trials and pre‐post interventional studies. Qualitative studies, observational studies, grey literature, book chapters, letters to the editor, conference abstracts, case reports, case series, reviews, non‐peer‐reviewed studies and studies not available in English were excluded.

### 2.3 Search Strategy

An electronic search on PubMed, EMBASE, CINAHL, and Cochrane Central databases was performed on 7 March 2024. Keywords for search strings were defined based on the population of interest (individuals with TMD), intervention (therapeutic exercise) and outcome (patient‐reported measures of activity, participation and QOL) which were combined using the ‘AND’ Boolean operator. Synonyms within each string were combined using the ‘OR’ operator.

Key search terms comprised a combination of Medical Subject Headings (MeSH terms), title terms, abstract terms, or text words and were adapted to fit within the format of each database. The search strategy and full search strings are outlined in the Appendix S1.

The following limiters were applied when available based on the database: English language, peer reviewed and human.

### 2.4 Selection of Studies

All studies identified from the search were imported to Endnote20 [12] to remove duplicates. Remaining studies were uploaded to Covidence [13] for screening. Four independent reviewers (AL, AP, LR and SS) screened titles and abstracts to identify all potentially relevant articles for further full‐text screening. Each study was independently screened by two separate reviewers. Any conflicts during the screening process were resolved through discussion among the four reviewers until there was consensus based on previously described eligibility criteria. If consensus could not be reached, the additional members of the research team were consulted (AD, RF). Reasons for exclusion following full‐text screening were recorded in Covidence. Inter‐rater agreement was measured using Cohen's Kappa.

### 2.5 Data Collection Process

One of two pairs of reviewers (AL, AP, LR, SS) independently extracted data from each study via a standardised Microsoft Excel template [14]. Each pair of reviewers discussed conflicts in extraction until consensus was reached. Collected data included: author, year, journal, study design, study setting/context, inclusion and exclusion criteria, number of participants and relevant participant demographics (e.g., age, sex), intervention characteristics, outcome measures and results relevant to this review's outcome of interest, main conclusions and limitations. Any missing data were marked ‘Not available (N/A)’.

### 2.6 Risk of Bias

A risk of bias assessment was performed using the Cochrane risk‐of‐bias tool v2 (RoB2) [15] for randomised control trials, the Risk of Bias in Non‐randomised Studies of Interventions (ROBINS‐I) for non‐randomised trials [16], and the National Institutes of Health, National Heart, Lung and Blood Institute (NIH NHLBI) Quality Assessment Tool for Before‐After (Pre‐Post) Studies with No Control Group [17] for pre‐post interventional studies. As per data extraction, the risk of bias assessment was performed by two pairs of independent reviewers.

The RoB2 tool assessed bias from five domains: the randomization process, deviations from intended interventions, missing outcome data, measurement of the outcome and selection of the reported result. The NIH NHLBI Pre‐Post tool assessed bias from seven categories: study aim, eligibility criteria, sample size, intervention, outcome measures, loss to follow‐up and statistical analysis. The ROBINS‐I assessed risk of bias from seven domains: confounding, selection of participants, intervention, deviations from intended interventions, missing data, outcomes and results. Each study was then given an overall risk of bias rating. Conflicts at this stage were resolved by discussion among the four reviewers until consensus was reached.

### 2.7 Data Analysis

Collected data were grouped according to outcome and analysed using a narrative synthesis approach. As the aim of this review was to determine the effect of an intervention, the primary focus of analysis was to explore within‐group data (i.e., comparing baseline to post‐intervention data within a group). A secondary analysis of between‐group data was then performed for studies comparing an intervention to control/placebo/alternate intervention.

The level of certainty of the evidence for each primary and secondary outcome was assessed as high, moderate, low, or very low using a modified Grading of Recommendations Assessment, Development and Evaluation (GRADE) approach [18, 19]. Quality of evidence for each outcome was assessed under five domains: risk of bias, inconsistency of findings, indirectness to the clinical question, imprecision of estimates and publication bias. Quality of evidence was then upgraded or downgraded accordingly. Studies that contained data for more than one outcome (e.g., activity and QOL) were included across multiple GRADE analyses.

## 3 Results

### 3.1 Study Selection

**FIGURE 1:**
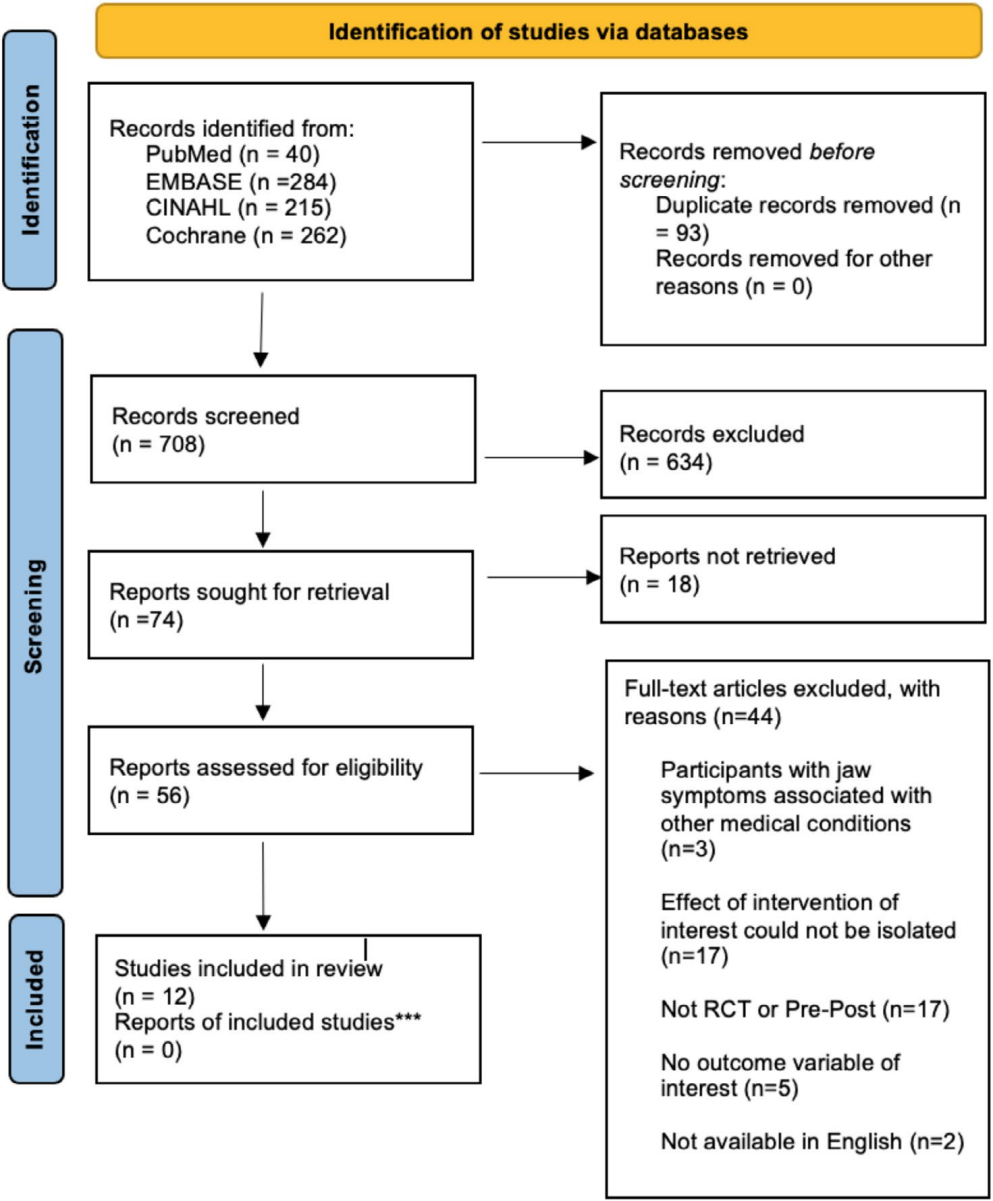
PRISMA flow diagram.

Figure 1 depicts the PRISMA flow diagram of this review. The initial search retrieved 708 studies. Following the removal of duplicates, title and abstract screening and full‐text screening, 12 studies were considered relevant and included in this review. Reasons for exclusion at full‐text screening stage are outlined in Appendix S2.

### 3.2 Participants

A total of 775 participants with TMD were included across the 12 studies. Table 1 outlines demographic data of participants.

**TABLE 1:** Study characteristics.

References	Country	Study design	Diagnostic criteria	TMD subgroup	Participants
de Almeida et al. 2023 [20]	Portugal	RCT	DC/TMD	DDwoR	Mandibular exercise therapy: *n* = 28 (mean age = 48.6 ± 14.3 years, 85% female) Arthrocentesis + Sodium Hyaluronate: *n* = 28 (mean age = 47.3 ± 18.3 years, 62% female)
Haketa et al. 2010 [21]	Japan	RCT	MRI	DDwR	Exercise therapy: *n* = 19 (mean age = 38.8 ± 15.2 years, 100% female) Splint: *n* = 25 (mean age = 38.6 ± 13.8 years, 84% female)
Kirschneck et al. 2013 [22]	Germany	Pre‐Post	“Suspected TMD Diagnosis”	Not specified	Cluster 1: *n* = 27 Cluster 2: *n* = 28 Cluster 3: *n* = 43 Mean age = 37.7 ± 13.7 years, 67% female^a^
Lindfors et al. 2020 [23]	Sweden	RCT	RDC/TMD	Not specified	Jaw exercise: *n* = 35 (mean age = 33.2 ± 18.7 years, 86% female) Stabilisation device: *n* = 33 (mean age = 32.7 ± 17.7 years, 79% female) Control: *n* = 29 (mean age = 40.5 ± 17.2 years, 72% female)
Magesty et al. 2021 [24]	Brazil	RCT	RDC/TMD	DDwR	Counselling + Jaw exercise: *n* = 36 Counselling: *n* = 36 Mean age = 22.8 ± 7.26 years, 69% female^a^
Maluf et al. 2010 [25]	Brazil	RCT	Helkimo Index	Not specified	Global postural re‐education: *n* = 12 (mean age = 30.00 ± 4.30 years, 100% female) Static stretching: *n* = 12 (mean age = 30.08 ± 7.07 years, 100% female)
Moleirinho‐Alves et al. 2021 [26]	Portugal Denmark	Pre‐Post	DC/TMD	Not specified	Therapeutic exercise: *n* = 15 (mean age = 26.9 ± 5.5 years, 86% female) Therapeutic exercise and Aerobic exercise: *n* = 15 (mean age = 26.0 ± 4.4 years, 86% female) Aerobic exercise: *n* = 15 (mean age = 24.9 ± 3.4 years, 86% female)
Moleirinho‐Alves et al. 2021 [27]	Portugal	Non‐RCT	DC/TMD	Not specified	Weekly strengthening/Coordinating exercises + Massage: *n* = 12 (mean age = 26.2 ± 5.7 years, 84% female) Weekly strengthening/Coordination exercises + Massage + Aerobic training: *n* = 12 (mean age = 26.0 ± 4.6 years, 84% female) Aerobic training: *n* = 12 (mean age = 25.8 ± 2.94 years, 84% female)
Ozmen et al. 2024 [28]	Turkey	RCT	DC/TMD	Muscle‐related TMD	Dry needling: *n* = 30 (mean age = 35.6 ± 9.9 years, 47% female) Face yoga: *n* = 30 (mean age = 35.6 ± 8.3 years, 37% female) Control: *n* = 30 (mean age = 35.6 ± 4.7 years, 53% female)
Seyhan et al. 2023 [29]	Turkey	RCT	DC/TMD	TMD of muscular origin	Core stability: *n* = 31 (mean age = 41.05 ± 15.0 years, 90% female) Control: *n* = 22 (mean age = 26.09 ± 8.52 years, 90% female)
Wahlund et al. 2015 [30]	Sweden	RCT	RDC/TMD	Not specified	Relaxation training: *n* = 31 (mean age = 16.50 ± 1.86 years, 97% female) Occlusal appliance: *n* = 22 (mean age = 16.3 ± 1.91 years, 94% female)
Wänman et al. 2020 [31]	Sweden	RCT	RDC/TMD	DDwR	Bite splint: *n* = 30 (mean age = 40.4 ± 17.0 years, 66% female) Home exercise: *n* = 30 (mean age = 38.5 ± 14.4 years, 80% female) Supervised exercise: *n* = 30 (mean age = 37.1 ± 14.1 years, 63% female)

Abbreviations: DC/TMD, Diagnostic Criteria for Temporomandibular Disorders; DDwoR, disc displacement without reduction; DDwR, disc displacement with reduction; RCT, randomised control trial; RDC/TMD, Research Diagnostic Criteria for Temporomandibular Disorders; TMD, temporomandibular disorder.

^a^ Individual group demographic data not presented, demographic data reported for entire study sample.

### 3.3 Study Characteristics

Table 2 summarises the characteristics of included studies. Of the 12 studies included in this review, nine were RCTs, two were pre–post studies and one was a non‐randomised trial.

**TABLE 2:** Intervention characteristics and results for primary outcome measures (activity, participation, quality of life).

References	Group	Intervention description	Intervention frequency and duration	Measurement of outcome	Results	
					Within group data	Between group data
					(Mean ± SD; unless otherwise stated)	
de Almeida et al. 2023 [20]	Mandibular exercise therapy	A set of home exercises including jaw opening and closing, ROM exercises and breathing exercises	10 reps 5 times per day in the same sequence for 1 month	OHIP‐14/56, where higher scores = greater impact on quality of life	*Functional limitation* Baseline: 2.6 ± 1.8 1 month: 1.4 ± 1.4 12 months: 1.5 ± 1.1 *Physical pain* Baseline: 6.4 ± 1.3 1 month: 4.9 ± 2.0 12 months: 4.5 ± 2.0 *Psychological discomfort* Baseline: 5.3 ± 1.7 1 month: 2.9 ± 2.2 12 months: 3.7 ± 2.1 *Physical disability* Baseline: 4.9 ± 1.8 1 month: 4.0 ± 2.1 12 months: 3.6 ± 2.4 *Psychological disability* Baseline: 4.2 ± 1.8 1 month: 2.9 ± 1.8 12 months: 3.2 ± 1.9 *Social* Baseline: 3.9 ± 2.0 1 month: 2.3 ± 1.9 12 months: 2.7 ± 1.9 *Handicap* Baseline: 3.0 ± 1.9 1 month: 1.9 ± 1.5 12 months: 2.3 ± 1.6	*Functional limitation* Baseline: *p* = 0.597 1 month: *p* = 0.600 12 months: *p* = 0.008* *Physical pain* Baseline: *p* = 0.116 1 month: *p* = 0.001* 12 months: *p* = 0.120 *Psychological discomfort* Baseline: *p* = 0.246 1 month: *p* = 0.253 12 months: *p* < 0.001* *Physical disability* Baseline: *p* = 0.270 1 month: *p* = 0.002* 12 months: *p* = 0.101 *Psychological disability* Baseline: *p* = 0.024 1 month: *p* = 0.029 12 months: *p* < 0.001* *Social* Baseline: *p* = 0.406 1 month: *p* = 0.253 12 months: *p* < 0.001* *Handicap* Baseline: *p* = 0.131 1 month: *p* = 0.033 12 months: *p* = 0.005*

Arthrocentesis + Sodium Hyaluronate (ASH)	5 mL of saline solution at 0.9% administered to distend joint space + intraoral mobilisation techniques post‐injection performed by physiotherapist	Saline was administered 5 times, intraoral techniques applied for 10 min afterwards		*Functional limitation* Baseline: 2.4 ± 2.0 1 month: 1.1 ± 1.1 12 months: 0.7 ± 0.7 *Physical pain* Baseline: 5.8 ± 1.4 1 month: 3.3 ± 1.2 12 months: 3.6 ± 1.4 *Psychological discomfort* Baseline: 4.6 ± 2.0 1 month: 2.2 ± 1.6 12 months: 1.7 ± 1.4 *Physical disability* Baseline: 5.4 ± 1.6 1 month: 2.3 ± 1.6 12 months: 2.5 ± 1.7 *Psychological disability* Baseline: 3.2 ± 1.8 1 month: 1.8 ± 1.5 12 months: 1.5 ± 1.0 *Social* Baseline: 3.3 ± 2.1 1 month: 1.2 ± 1.4 12 months: 1.4 ± 2.1 *Handicap* Baseline: 2.2 ± 1.4 1 month: 1.0 ± 0.8 12 months: 1.1 ± 1.0	
Haketa et al. 2010 [21]	Exercise therapy	Manual jaw opening exercises conducted by participants themselves + verbal explanation of their condition	Position held for 30 s, repeated 3 times per set. Sets repeated 4 times per day for 8 weeks	Limitation of Daily Functions for the TMD Questionnaire/50	(Median, IQR) Baseline: 24, 21–27 4 weeks: 20, 17–22 (*p* < 0.001*) 8 weeks: 18, 15–20 (*p* < 0.001*)	No information
	Splint therapy	Maxillary stabilisation appliance + verbal explanation of their condition	Participants instructed to wear splint at night while sleeping for 8 weeks		(Median, IQR) Baseline: 27, 22–29 4 weeks: 23, 21–26 (*p* < 0.001*) 8 weeks: 22, 18–26 (*p* < 0.001*)	
Kirschneck et al. 2013 [22]	Muscle relaxation	Audio tape instructions on progressive muscle relaxation	Participants advised to listen to as often as possible over 3 months	PI‐A4 (Pain‐Related Interference)/5	(Median, IQR) Pre‐treatment: 2.0, 1.5–3.0 3 months: 2.0, 1.5–3.0*	No information
Lindfors et al. 2020 [23]	Therapeutic jaw exercises	Relaxation, ROM and resistance exercises and stretching	10 reps per exercise and stretching 2–3 times per day for 3 months	JFLS/20, where higher scores = greater limitation	(Median, Range) Baseline: 2.0, 0–5.7 3 months: 0.4, 0–5.7	Difference between therapeutic jaw exercise vs. no treatment: *p* = 0.008*
	Oral stabilisation appliance	Hard acrylic resin stabilisation appliance to achieve canine guidance	Patient were instructed to wear every night for 3 months		(Median, Range) Baseline: 1.1, 0–6.3 3 months: 0.5, 0–6.0	
	No treatment	No treatment received	No data		(Median, Range) Baseline: 1.5, 0–8.3 3 months: 1.6, 0–6.1	
Magesty et al. 2021 [24]	Jaw exercises + counselling	Opening and closing exercises +/− resistance, diet limited to soft foods, application of heat and cold therapy for pain management, posture education, decrease caffeine intake.	20 reps, repeated 3 times a day for 30 days	OHIP‐14/56, where higher scores = greater impact on quality of life	(Mean) *Functional Limitation* Baseline: 0.36 30 days: 0.09* *Physical Pain* Baseline: 3.06 30 days: 1.00* *Psychological Discomfort* Baseline: 2.45 30 days: 0.64* *Physical Pain Disability* Baseline: 0.61 30 days: 0.15* *Psychological Incapacity* Baseline: 1.79 30 days: 0.39* *Social Incapacity* Baseline: 0.88 30 days: 0.09* *Disability* Baseline: 0.45 30 days: 0.09 (*p* = 0.066) *Total* Baseline: 9.61 30 days: 2.45*	*Functional Limitation*: *p* = 0.52 *Physical Pain*: *p* = 0.004* *Psychological Discomfort*: *p* < 0.001* *Physical Pain Disability*: *p* = 0.398 *Psychological Incapacity*: *p* < 0.001* *Social Incapacity*: *p* = 0.029* *Disability*: *p* = 0.088 *Total*: *p* < 0.001*

Counselling only	Diet limited to soft foods, application of heat and cold therapy for pain management, posture education, decrease caffeine intake.		OHIP‐14/56, where higher scores = greater impact on quality of life	(Mean) *Functional Limitation* Baseline: 0.30 30 days: 0.36 (*p* = 0.458) *Physical Pain* Baseline: 2.73 30 days: 1.76* *Psychological Discomfort* Baseline: 1.58 30 days: 1.30 (*p* = 0.259) *Physical Pain Disability* Baseline: 0.70 30 days: 0.39 (*p* = 0.109) *Psychological Incapacity* Baseline: 1.15 30 days: 0.97 (*p* = 0.230) *Social Incapacity* Baseline: 0.42 30 days: 0.18* *Disability* Baseline: 0.33 30 days: 0.27 (*p* = 0.458) *Total* Baseline: 7.21 30 days: 5.24*	
Maluf et al. 2010 [25]	Global posture re‐education	Participants maintained 2 different postures to stretch both posterior and anterior muscle chains	Each posture was held for 15 min, in weekly sessions for 2 months	Sleep restriction reported on VAS scale 0 = no difficulty, 10 = very difficult	Baseline: 5.69 ± 3.45 Post‐treatment: 3.08 ± 3.05 (*p* = 0.100) 8 week follow up: 3.22 ± 3.31 (*p* = 0.127)	No information
	Static stretching	Stretching performed for cervical spine, head, upper limbs and mandibular muscles.	Each stretching position was held for 30 s, repeated bilaterally 3 times after 10 s rest, once a week for 2 months		Baseline: 4.65 ± 3.36 Post‐treatment: 3.00 ± 2.38 (*p* = 0.389) 8 week follow up: 3.02 ± 2.33 (*p* = 0.400)	
Moleirinho‐Alves et al. 2021 [26]	Strengthening and coordination exercises + massage	Massage of mandibular with isotonic strengthening exercises performed by the same physiotherapist	10 reps of each exercise and massage for 30 min once per week for 8 weeks	OHIP‐14/56, where higher scores = greater impact on quality of life	(Mean, 95% CI) Baseline 1: 18.7, 12.76 to 24.71 Baseline 2: 18.9, 13.16 to 24.71 58 days post: 5.2, 1.55 to 8.85* 8–12 weeks post: 5.2, 1.55 to 8.85	No Information
	Strengthening and coordination exercises + massage + aerobic training	Massage of mandibular with isotonic strengthening exercises performed by physiotherapist + aerobic cycle ergometer training sessions all supervised by same physiotherapist	10 reps of each exercise for 30 min and massage once per week for 8 weeks + aerobic cycling performed 2 times per week for 8 weeks		(Mean, 95% CI) Baseline 1: 16.5, 10.56 to 22.51 Baseline 2: 16.5, 10.76 to 22.31 58 days post: 0.7, −2.92 to 4.38* 8–12 weeks post: 0.7, −2.92 to 4.38	
	Aerobic training	Aerobic cycle ergometer training sessions supervised by same physiotherapist	30 min 2 times per week for 8 weeks		(Mean, 95% CI) Baseline 1: 10.8, 4.83 to 16.77 Baseline 2: 11.5, 5.69 to 17.24 58 days post: 9.6, 5.95 to 13.25 8–12 weeks post: 9.6, 5.96 to 13.25	
Moleirinho‐Alves et al. 2021 [27]	Strengthening and coordination exercises + massage	Massage of mandibular muscles with isotonic strengthening exercises performed by same physiotherapist	10 reps of each exercise and massage for 30 min once per week for 8 weeks	HIT‐6/78, where higher scores = more impact	(Mean, 95% CI) Baseline 1: 66.9, 56.72 to 77.15 Baseline 2: 67.6, 57.64 to 77.56 48 h post: 50.7, 38.01 to 63.46* 8–12 weeks follow‐up: 55.1, 43.00 to 67.27*	No information
	Strengthening and coordination exercises + massage + aerobic training	Massage of mandibular muscles with isotonic strengthening exercises performed my physiotherapist + aerobic cycle ergometer training sessions all supervised by same physiotherapist	10 reps of each exercise for 30 min and massage 1x per week for 8 weeks + aerobic cycling performed 2 times per week for 8 weeks		(Mean, 95% CI) Baseline 1: 62.9, 52.72 to 73.15 Baseline 2: 63.7, 53.71 to 73.62 48 h post: 49.5, 36.81 to 62.26* 8–12 weeks follow‐up: 51.7, 39.53–63.80*	
	Aerobic training	Aerobic cycle ergometer training sessions supervised by same physiotherapist	30 min 2 times per week for 8 weeks		(Mean, 95% CI) Baseline 1: 62.2, 51.99 to 72.42 Baseline 2: 62.5, 52.58 to 72.49 48 h post: 59.3, 46.54 to 71.99 8–12 weeks follow‐up: 59.8, 47.67 to 71.94	
Ozmen et al. 2024 [28]	Face yoga	Applied to the face, jaw and neck regions	8 exercises repeated 10 times each, conducted 3 times per week for 6 weeks	PSQI/21, with scores ≥ 5 indicating clinically significant poor sleep quality	Pre‐treatment: 10.60 ± 2.19 Post‐treatment: 10.17 ± 2.04*	Pre‐treatment: *p* = 0.014* Post‐treatment: *p* = 0.021*
	Dry needling	Stainless steel needles 13 mm × 0.25 mm utilised in master and temporal muscles, rotated clockwise upon insertion	Left in for 10 min, reversed counterclockwise and left for a further 10 min. Conducted 3 times per week for 6 weeks		Pre‐treatment: 9.83 ± 3.66 Post‐treatment: 8.97 ± 2.89*	
	Control	Soft food diet, heat application and analgesic medication	Advice given once at beginning of study		Pre‐treatment: 8.40 ± 2.59 Post‐treatment: 8.40 ± 2.39 (*p* = 0.999)	
Seyhan et al. 2023 [29]	Core stability exercises + Patient education + Rocabado exercises	5 exercises to target core stability controlled by a physiotherapist, patient education about condition and exacerbating factors, the 6 Rocabado exercises	Core stability was performed 3 times per week (once in person and twice online) for 6 weeks. Patient education given once at beginning of study. Rocabado exercises performed 6 reps each, 6 times per day for 6 weeks	JFLS/20, where higher scores = greater limitation OHIP‐14/56, where higher scores = greater impact on quality of life	*JFLS/20*: Pre‐treatment: 39.1 ± 20.9 Post‐treatment: 31.8 ± 21.45* *OHIP‐14*: Pre‐treatment: 15.9 ± 6.47 Post‐treatment: 11.05 ± 5.73*	*JFLS‐20*: Pre‐treatment: *p* = 0.903, Cohen's *d* = 0.01 Post‐treatment: *p* = 0.685, Cohen's *d* = 0.06 *OHIP‐14*: Pre‐treatment: *p* = 0.776, Cohen's *d* = 0.04 Post‐treatment: *p* = 0.871, Cohen's *d* = 0.02
	Patient education + Rocabado exercises	Patient education about condition and exacerbating factors, the 6 Rocabado exercises	Patient education given once at beginning of study. Rocabado exercises performed 6 reps each, 6 times per day for 6 weeks		*JFLS‐20*: Pre‐treatment: 48.1 ± 43.5 Post‐treatment: 31.8 ± 29.3* *OHIP‐14*: Pre‐treatment: 16.6 ± 10.76 Post‐treatment: 12.75 ± 10.07*	
Wahlund et al. 2015 [30]	Relaxation training	Relaxation techniques performed by a trained therapist, in addition to a home‐training program with manual and audio instructions	8 weekly in‐person sessions of 45 min, plus to 15–20 min a day of home training per day for 3 months	School absence due to TMD pain per month Scale of 0 to 31, where higher scores = more days absent	Pre‐treatment: 1.2 ± 2.1 Post‐treatment: 0.5 ± 1.0 Follow‐up: 0 ± 0 *Phase 2* ^a^ Post‐2: 0 ± 0 Follow up: 0 ± 0	No information
	Occlusal appliance	Therapist administered a stabilisation splint placed in the maxilla	Advised to use splint every night for 6 months		Pre‐treatment: 0.3 ± 0.8 Post‐treatment: 0.2 ± 0.8 Follow‐up: 0.2 ± 0.5 *Phase 2* ^a^ Post‐2: 0.2 ± 0.7 Follow up: 0.5 ± 1.2	
Wänman et al. 2020 [31]	Supervised exercise therapy	Warm up with heat therapy, jaw opening‐closing exercises, jaw opening and protrusion against resistance	5 min warm up, 6 min of jaw opening‐closing exercises and 4 min of resistance exercises repeated in 10 sessions over 3 months	JFLS/20, where higher scores = greater limitation NDI/50, where higher scores = greater disability	*JFLS/20*: Pre‐treatment: 18.5 ± 15.5 Post‐treatment: 14.0 ± 13.0 *NDI/50*: Pre‐treatment = 10.0 ± 10.8 Post‐treatment = 7.9 ± 7.8 (*p* = 0.016*)	No information
	Bite splint therapy	A resilient bite splint, 4 mm thick of BIOPLAST material	Participants instructed to wear when sleeping at night for 3 months		*JFLS/20*: Pre‐treatment: 16.3 ± 18.2 Post‐treatment: 16.0 ± 13.1 *NDI/50*: Pre‐treatment = 8.1 ± 7.6 Post‐treatment = 6.8 ± 5.9	
	Home exercise therapy	Home exercise regime of jaw opening and closing exercises, isometric opening exercises and protrusion against resistance	Jaw opening and closing exercises repeated for 5 min after every meal. Isometric and resistance exercises held for 10 s × 10 reps daily for 3 months		*JFLS/20*: Pre‐treatment: 18.9 ± 33.3 Post‐treatment: 14.6 ± 16.4 *NDI/50*: Pre‐treatment = 11.4 ± 10.7 Post‐treatment = 10.3 ± 12.4	

Abbreviations: HIT‐6, Headache Impact Test‐6; JFLS‐20, Jaw Functional Limitation Scale‐20; NDI, Neck Disability Index; OHIP‐14, Oral Health Impact Profile‐14; PI‐A4, Pain Inventory‐A4; PSQI, Pittsburgh Sleep Quality Index; TMD, Temporomandibular Disorder; VAS, Visual Analog Scale.

^a^ Phase 2 of this study included sequential treatment for non‐responders.

* *p* < 0.05 suggesting statistical significance.

### 3.4 Interventions

The interventions evaluated across the 12 studies in this review are presented in Table 2. Seven studies included at least one intervention group where exercise was administered independent of any other intervention [20, 22, 23, 25, 28, 30, 31], while the remaining five comprised groups where exercise was delivered in conjunction with other interventions such as patient education, massage and counselling [21, 24, 26, 27, 29]. With respect to the therapeutic exercise intervention, seven of the 12 studies evaluated the effects of jaw strengthening exercises [23, 24, 26, 27, 29, 31]. Six studies utilised jaw range of motion exercises [20, 21, 23, 24, 25, 31], while four studies included relaxation training [22, 23, 30] and “face yoga” [28], respectively. Forms of global exercise that did not directly involve the jaw included aerobic exercise [26, 27], posture re‐education [25], core stability exercises [29] and breathing techniques [20]. Seven studies compared the effects of therapeutic exercise to other management approaches, including oral stabilisation appliances, dietary changes, saline injections and dry needling [20, 21, 23, 24, 28, 30, 31].

### 3.5 Outcomes

Table 2 outlines the specific measures used to capture the primary activity, participation and QOL outcomes in this review. Seven studies provided outcome data for activity limitations, measured using the Jaw Functional Limitation Scale 20 (JFLS‐20, *n* = 3) [23, 29, 31], Limitation of Daily Functions for the TMD Questionnaire (LDFQ, *n* = 1) [21], Pittsburgh Sleep Quality Index (PSQI, *n* = 1) [28] and a Visual Analogue Scale (VAS) for pain‐related interference (*n* = 1) and sleep limitations (*n* = 1) [22, 25]. Two studies provided outcome data for participation restrictions, via reported days absent from school (*n* = 1) [30] and the Headache Impact Test questionnaire (HIT‐6, *n* = 1) [27]. Quality of Life (QoL) was assessed in five studies using the Oral Health Impact Profile 14 (OHIP‐14, *n* = 4) [20, 24, 26, 29] and Neck Disability Index (NDI, *n* = 1) [31].

Eight studies also provided data pertaining to secondary outcomes (Table 3). Of these, all reported data relating to pain intensity, measured using a VAS (*n* = 5) [20, 21, 23, 26, 28], Numerical Rating Scale (NRS, *n* = 2) [27, 30], or a Likert scale (*n* = 1) [22]. Three studies reported data relating to jaw range of motion, namely maximal mouth opening (mm) [20, 21, 30].

**TABLE 3:** Results for secondary outcome measures pertaining to the body structures and functions domain.

Citation	Group	Measurement of outcome	Results	
			Within group data (Mean ± SD; unless otherwise stated)	Between group data
de Almeida et al. 2023 [20]	Mandibular exercise therapy	Pain VAS/10, where higher scores = greater pain Mouth‐opening range (mm)	*VAS/10*: Baseline: 6.8 ± 2.3 1 month: 3.0 ± 2.5 *Pain‐free opening*: Baseline: 33.6 ± 6.2 1 month: 37.2 ± 5.9 12 months: 37.5 ± 5.0 Difference from baseline At 1 month: 3.7 ± 3.1 At 12 months: 3.9 ± 3.4 *Maximum unassisted opening*: Baseline: 37.1 ± 5.5 1 month: 39.5 ± 5.1 12 months: 39.8 ± 4.6 Difference from baseline At 1 month: 2.4 ± 2.5 At 12 months: 2.4 ± 2.5	*VAS/10*: Baseline: *p* = 0.648 1 month: *p* = 0.328 12 months: *p* = 0.355 *Pain‐free opening*: Baseline: *p* = 0.378 1 month: *p* = 0.254 12 months: *p* = 0.125 Difference from baseline At 1 month: *p* = 0.007* At 12 months: *p* = 0.016* *Maximum unassisted opening*: Baseline: *p* = 0.239 1 month: *p* = 0.142 12 months: *p* = 0.101 Difference from baseline At 1 month: *p* = 0.003* At 12 months: *p* = 0.008*
	Arthrocentesis + Sodium Hyaluronate (ASH)		*VAS/10*: Baseline: 6.7 ± 2.2 1 month: 3.3 ± 1.2 12 months: 2.4 ± 2.1 *Pain‐free opening*: Baseline: 32.6 ± 5.9 1 month: 38.8 ± 4.5 12 months: 39.5 ± 4.7 Difference from baseline At 1 month: 6.2 ± 4.4 At 12 months: 6.9 ± 4.5 *Maximum unassisted opening*: Baseline: 36.0 ± 4.9 1 month: 41.4 ± 4.0 12 months: 41.7 ± 4.2 Difference from baseline At 1 month: 5.4 ± 3.2 At 12 months: 5.6 ± 3.7	
Haketa et al. 2010 [21]	Exercise therapy	Pain VAS/100, where higher scores = greater pain Mouth‐opening range (mm)	*Pain VAS/100*: Baseline: 63.1 ± 21.4 4 weeks: 33.1 ± 26.8 8 weeks: 21.3 ± 26.4 *Mouth‐opening range (mm)*: Without pain: Baseline: 26.5 ± 5.6 4 weeks: 35.1 ± 5.6 8 weeks: 37.8 ± 6.4 (*p* < 0.001*) With pain: Baseline: 32.3 ± 5.5 4 weeks: 39.3 ± 4.9 8 weeks: 41.0 ± 5.4 (*p* < 0.001*)	*Pain VAS/100*: *p* < 0.001* *Mouth‐opening range (mm)*: Without pain: *p* < 0.001* With pain: *p* < 0.001*
	Splint therapy		*Pain VAS/100*: Baseline: 58.9 ± 28.2 4 weeks: 43.5 ± 27.1 8 weeks: 36.5 ± 28.7 *Mouth‐opening range (mm)*: Without pain: Baseline: 25.7 ± 8.2 4 weeks: 29.6 ± 7.8 8 weeks: 31.6 ± 7.6 With pain: Baseline: 30.3 ± 7.7 4 weeks: 33.0 ± 6.4 8 weeks: 35.0 ± 5.8	
Kirschneck et al. 2013 [22]	Muscle relaxation	P1‐A1 (pain intensity)/6, where a higher number = greater pain	(Median, IQR) Pre‐treatment: 3.0, 2.0–3.0 Post‐treatment: 2.5, 2.0–3.0	*p* < 0.001*
Lindfors et al. 2020 [23]	Therapeutic jaw exercises, comprising jaw resistance and mobility exercises	Pain VAS/10, where higher scores = greater pain	No values reported	Between therapeutic jaw exercise + no treatment *p* < 0.001*
	Oral stabilisation appliance			
	No treatment			
Moleirinho‐Alves et al. 2021 [26]	Strengthening and coordination exercises + massage	Pain VAS/10, where higher scores = greater pain	(Mean, 95% CI) Baseline 1: 5.5, 4.86 to 6.20 Baseline 2: 5.3, 4.56 to 5.98 58 days post: 0.3, −0.47 to 1.13* 8–12 weeks post: 0.5, −0.25 to 1.32	Baseline 1: *p* = 0.86
	Strengthening and coordination exercises + massage aerobic training		(Mean, 95% CI) Baseline 1: 6.0, 5.33 to 6.67 Baseline 2: 5.9, 5.16 to 6.58 58 days post: 0.0, −0.80 to 0.80* 8–12 weeks post: 0.0, −0.80 to 0.80	
	Aerobic training		(Mean, 95% CI) Baseline 1: 5.5, 4.86 to 6.20 Baseline 2: 5.3, 4.62 to 6.04 58 days post: 3.3, 2.54 to 4.13* 8–12 weeks post: 4.1, 3.35 to 4.92	
Moleirinho‐Alves et al. 2021 [27]	Strengthening and coordination exercises + massage	Pain NRS/10, where higher scores = greater pain	(Mean, 95% CI) Baseline 1: not reported Baseline 2: 5.4 48 h post: 0.3, 95% CI = −0.40 to 1.068* 8–12 weeks follow‐up: 0.5, 95% CI = −0.256 to 1.256	Difference between all groups between baseline 1 and 2 (*p* = 0.53) Difference between all groups between baseline 2 and post intervention (*p* < 0.001) 48 h post: 49.5, 95% CI = 36.808–62.259* 8–12 weeks follow‐up: 51.7, 95% CI = 39.532–63.802
	Strengthening and coordination exercises + massage + aerobic training		Baseline 1: not reported Baseline 2: 5.8 48 h post: 0.1, 95% CI = −0.734 to 0.734* 8–12 weeks follow‐up: 0.0, 95% CI = −0.756 to 0.756	
	Aerobic training		Baseline 1: not reported Baseline 2: 5.2 48 h post: 2.9*	
Ozmen et al. 2024 [28]	Face yoga	Pain VAS/10, where higher scores = greater pain	Pre‐treatment: 7.70 ± 0.65 Post‐treatment: 5.87 ± 1.17	Pre‐treatment: *p* = 0.160 Post = treatment: *p* = 0.001*
	Dry needling		Pre‐treatment: 7.63 ± 0.61 Post‐treatment: 4.43 ± 0.77	
	Control		Pre‐treatment: 7.93 ± 0.64 Post‐treatment: 7.27 ± 0.64	
Wahlund et al. 2015 [30]	Relaxation training	Pain NRS/10, where higher scores = greater pain Mouth opening range (mm)	*NRS/10*: Pre‐treatment: 5.4 ± 1.9 Post‐treatment: 4.4 ± 1.8 Follow‐up: 2.8 ± 1.9 Phase 2^a^ Post‐2: 4.6 ± 2.3 Follow up: 4.0 ± 2.6 *Mouth opening range*: Pre‐treatment: 44.9 ± 10.9 Post‐treatment: 45.3 ± 9.2 Follow‐up: 52.4 ± 7.8 Phase 2^a^ Post‐treatment: 46.1 ± 7.2 Follow‐up: 48.7 ± 6.9	Not reported
	Occlusal appliance		*NRS/10*: Pre‐treatment:5.5 ± 2.0 Post‐treatment: 3.7 ± 2.0 Follow‐up: 2.8 ± 1.6 *Phase 2* ^a^ Post‐2: 3.6 ± 1.8 Follow up: 3.6 ± 2.2 *Mouth opening range*: Pre‐treatment: 40.2 ± 8.6 Post‐treatment: 43.1 ± 8.0 Follow‐up: 46.4 ± 8.4 *Phase 2* ^a^ Post‐treatment: 49.0 ± 10.0 Follow‐up: 46.1 ± 9.0	

Abbreviations: NRS, Numerical rating scale; PI‐A1, Pain Inventory‐A1; VAS, Visual analog scale.

^a^ Phase 2 of this study included sequential treatment for non‐responders.

* *p* < 0.05 suggesting statistical significance.

### 3.6 Risk of Bias

Table 4 reports the overall results of the risk of bias assessment. All studies were considered to have a high risk of bias, primarily due to deviations from the intended interventions and/or a high risk of bias in the measurement domain. It is important to note for the latter that the outcome measures used were all participant‐reported questionnaires, due to the nature of the primary outcomes of this review (activity limitations, participation restrictions and QOL) and thus prone to bias, particularly in the absence of participant blinding [32].

**TABLE 4:** Risk of bias assessment.

References	Domain 1: Risk of bias arising from the randomisation process	Domain 2: Risk of bias due to deviations from the intended interventions (effect of assignment to intervention)	Domain 3: Missing outcome data	Domain 4: Risk of bias in measurement of the outcome	Domain 5: Risk of bias in selection of the reported result	Overall risk of bias
de Almeida et al. 2023 [20]	Low	Some concerns	Low	High	Low	High
Haketa et al. 2010 [21]	Low	Some concerns	High	High	Low	High
Kirschneck et al. 2013 [22]	^a^					Poor
Lindfors et al. 2020 [23]	Low	High	Some concerns	High	Low	High
Magesty et al. 2021 [24]	Low	Some concerns	Low	High	Low	High
Maluf et al. 2010 [25]	Low	Some concerns	Low	High	Low	High
Moleirinho‐Alves et al. 2021 [26]	^a^					Poor
Moleirinho‐Alves et al. 2021 [27]	^b^					Very Serious Concern
Ozmen et al. 2024 [28]	Low	Some concerns	Low	High	Low	High
Seyhan et al. 2023 [29]	Low	Some concerns	Low	High	Low	High
Wahlund et al. 2015 [30]	Low	Some concerns	Low	High	Low	High
Wänman et al. 2020 [31]	Low	Some concerns	Low	High	Low	High

^a^ Pre‐post study used NIH NHLBI pre‐post tool; therefore, only overall risk of bias stated (Appendix S3).

^b^ Non‐Randomised used ROBINS‐I tool; therefore, only overall risk of bias stated (Appendix S4).

### 3.7 Main Findings

Table 2 presents a summary of findings from each study, including within and between group data.

#### 3.7.1 Effect of Therapeutic Exercise on Activity

##### 3.7.1.1 Activities of Daily Living

Five studies explored the effect of therapeutic exercise on individuals' ability to perform activities of daily living [21, 22, 23, 29, 31]. Of these, three found a significant improvement in activity following the therapeutic exercise intervention [21, 22, 23], while the remaining two reported no difference [29, 31]. Key differences between the studies included the type of exercise used and the method of exercise delivery (i.e., home vs. supervised).

The three studies which reported a significant improvement in activity following exercise utilised programmes comprising localised strengthening, mobility and relaxation exercises [21, 22, 23]. One study compared the effects of a combined home‐based jaw resistance and mobility programme over a 3‐month period to a no‐treatment control and a stabilisation appliance group [23]. The exercise group showed a significant decrease in JFLS‐20 scores post‐intervention compared to the no‐treatment control group. Another study investigated the effect of manual mouth opening exercises compared to an occlusal splint [21]. When assessed at four –and 8‐weeks post‐intervention, a significant improvement in activity was observed in both groups; no statistical between‐group comparison was conducted. Lastly, a no‐comparator pre‐post study found a significant improvement in pain‐related activity levels following a 3‐month home‐based relaxation program [22]. The effect of the relaxation training was measured using a custom‐developed scale to rate the interference of pain on activity levels. The authors reported a significant improvement following the programme, though no statistical evidence was provided.

The two studies that reported no significant improvements in activity utilised home exercise programmes, supervised exercise therapy and core stability combined with Rocabado exercises [29, 31]. One study compared a 3‐month home‐based jaw mobility and resistance programme with a similar supervised programme and a bite stabilisation appliance [31]. No group reached a level of significance in JFLS‐20 scores post‐intervention. Another study examined a combined core stability, patient education and Rocabado exercise programme versus patient education and Rocabado exercise alone [29]. Neither group showed significant improvement in JFLS‐20 scores, and there was no difference between groups.

##### 3.7.1.2 Sleep

Two studies evaluated the effect of therapeutic exercise on sleep, with conflicting results. The first study compared ‘face yoga’ to dry needling and a control group over 6 weeks [28]. The face yoga and dry needling groups both showed significant improvement in sleep quality measured by the Pittsburgh Sleep Quality Index (PSQI). The control group, which received patient education on other management strategies, showed no significant change post‐intervention. The second study compared global posture re‐education with static stretching [25]. Neither intervention resulted in significant improvement in sleep restriction, and there was no difference between the groups.

#### 3.7.2 Effect of Therapeutic Exercise on Participation

##### 3.7.2.1 School Absence

One study compared the impact of 8 weeks of combined supervised and home‐based relaxation exercises to occlusal appliance therapy on school absenteeism in adolescents with TMD [30]. While relaxation exercises suggested a reduction in absences, the difference was not statistically significant in either group.

##### 3.7.2.2 Participation in Daily Life

One study assessed the impact of an 8‐week supervised aerobic exercise program (cycle ergometry), with or without jaw strengthening exercises and masticatory muscle massage, on interference of their headaches with daily activities using the HIT‐6 [27]. While participants who engaged in aerobic exercise alone reported decreased HIT‐6 scores, this improvement was only statistically significant when combined with jaw resistance exercises and massage interventions or when jaw resistance exercises and massage were performed independent of aerobic training. Improvements were sustained at the 8–12 week follow‐up.

#### 3.7.3 Effect of Therapeutic Exercise on QOL

The effect of therapeutic exercise on the QOL of individuals with TMD was evaluated in five studies. Of these, four found significant improvements in QOL post‐exercise intervention [24, 26, 29], while one did not report a significant difference [20].

The studies which found a significant improvement in QOL included jaw mobility, jaw strengthening and core stability exercises. Magesty et al. [24] assessed the effect of a 30‐day home‐based jaw mobility and resistance programme plus counselling (diet, pain management and postural education) compared to counselling alone. While both groups demonstrated significant improvements in OHIP‐14 scores post‐intervention, counselling alone only significantly improved the pain and social disability subscales of the OHIP‐14. When combined with exercise, six of seven subscales of the OHIP‐14 showed significant improvement. The second study evaluated the effect of jaw mobility and resistance exercises (home‐based or supervised) on NDI scores compared to a bite splint [31]. While the home and supervised exercise approaches both decreased NDI scores, only the supervised exercise group showed a significant improvement post‐intervention. The statistical difference between home and supervised exercise was not directly compared. The third study assessed the addition of supervised core stability exercises (e.g., plank, bridge, bird dog) into a TMD management protocol including education and Rocabado exercises [29]. After 6 weeks, significant improvements in OHIP‐14 scores were observed in both groups. No significant differences were observed between groups. Lastly, Moleirinho‐Alves et al. [26] used the OHIP‐14 questionnaire to evaluate 8 weeks of supervised aerobic exercise (cycle ergometry) alone and combined with jaw strengthening exercises and massage. Aerobic exercise alone did not significantly improve OHIP‐14 scores, but significant improvements were observed when combined with massage and jaw exercises.

The remaining study found daily home‐based jaw mobility and breathing exercises for 4 weeks decreased OHIP‐14 scores in all domains, although the significance of these findings was not commented on [20].

#### 3.7.4 Effect of Therapeutic Exercise on Body Functions and Structures

##### 3.7.4.1 Pain Intensity

Eight studies evaluated the effect of therapeutic exercise on pain. Of these, two compared supervised aerobic exercise programs alone (cycle ergometry) with aerobic exercise plus jaw resistance and mobility exercises and massage [26, 27]. The intervention across both studies was the same. The first study found that aerobic exercise alone did not significantly improve jaw pain [26], while the second study found the aerobic program did significantly improve headache intensity [27]. In both studies, a significant improvement in pain (jaw and headache) was observed when aerobic exercise was combined with targeted jaw exercises and massage.

Two studies in this review examined the effect of jaw mobility exercises on jaw pain intensity. Haketa et al. compared the effect of passive mouth opening exercises on jaw pain to an occlusal splint and found significant improvements in pain intensity in both groups at four and 8 weeks [21]. The exercise group had the greatest reduction in pain at 8 weeks compared to baseline (−41.8 mm VAS, vs. −22.4 mm in the splint group), although this between‐group difference was not significant. De Almeida et al. [20] compared the effect of jaw mobility exercises on jaw pain intensity to an Arthrocentesis & Sodium Hyaluronate (ASH) injection. Neither intervention demonstrated a significant within‐group improvement pre‐ to post‐intervention, though this study reported a significant between‐group difference post‐intervention favouring the ASH injection.

Another two studies investigated the effect of relaxation exercises on jaw pain intensity. One compared the effects of therapist‐guided relaxation training with occlusal appliance therapy on jaw pain and found no significant improvements in either group [30]. The other was a pre‐post study where the authors created a customised pain‐intensity Likert scale to quantify the effect of unsupervised muscle relaxation exercises on jaw pain. A significant improvement in pain was observed post‐intervention [22].

The remaining two studies explored the effect of face yoga or a combined jaw resistance and mobility program on jaw pain, respectively. Ozmen et al. [28] compared the effect of a home‐based face yoga program, in which ROM and massage techniques were applied to the face, jaw and neck, to dry needling and a no‐intervention control. Post‐intervention, a significant improvement in pain intensity was observed in the face yoga group and, to an even greater degree, the dry needling group. No change was seen in the control group. The final study compared a therapeutic exercise program comprising jaw resistance and mobility exercises with an oral stabilisation appliance and a no‐treatment group [23]. There was a significant decrease in pain intensity (VAS) in the jaw exercise group compared to the no‐treatment group.

##### 3.7.4.2 Jaw Range of Motion

Three studies explored the effect of therapeutic exercise on jaw ROM. Of these, two studies assessed the effectiveness of jaw mobility exercises on mouth opening ROM [20, 21]. The first study compared 8 weeks of home‐based jaw mobility exercises to splint therapy, finding significant improvements in pain‐free and maximal mouth opening ROM for both interventions, without indicating which was more effective [21]. The second study compared 1 month of home‐based jaw mobility exercises to an ASH injection [20]. Improvements in both pain‐free and maximal mouth opening range were observed in the exercise and ASH groups post‐intervention though were not significant.

One study compared supervised and home‐based relaxation exercises to an occlusal appliance [30]. No significant improvement in mouth opening range post‐intervention was demonstrated in either group.

#### 3.7.5 Certainty of Evidence

Table 5 outlines the certainty of evidence for each outcome. The certainty of evidence regarding the effect of therapeutic exercise on all outcomes was considered very low due to imprecision and/or risk of bias concerns.

**TABLE 5:** GRADE quality assessment for each outcome.

Outcome	Intervention	No. of studies (No. of participants)	Types of studies	Downgrading across each GRADE domain with reasons					Overall certainty
				Risk of bias	Inconsistency	Indirectness	Imprecision	Publication bias	
Activity limitations	Therapeutic exercise	7 (497)	RCT	*Very Serious (−2)*: These studies were considered to have a high risk of bias, due to outcome measurement (blinding) concerns, deviations from the intended interventions and/or reporting concerns	*Not serious (−0)*: No serious inconsistencies in findings were found across studies which could not be accounted for by differences in study populations, interventions, comparison/controls or outcomes	*Not serious (−0)*: No notable variations in population, intervention, or comparator were identified that were likely to have a meaningful impact on the outcomes	*Serious (−1)*: The cumulative sample size across these studies was greater less than *n* = 400, however, some or all of the studies did not provide adequate indication of effect	*Not suspected (−0)*: No indication of publication bias; the comprehensive search strategy successfully identified randomised controlled trials reporting both favourable and unfavourable results	*Very low* ⊕◯◯◯
Participation restrictions	Therapeutic exercise	2 (100)	RCT, pre‐post	*Very Serious (−2)*: These studies were considered to have a high risk of bias, due to outcome measurement (blinding) concerns	*Not serious (−0)*: No serious inconsistencies in findings were found across studies which could not be accounted for by differences in study populations, interventions, comparison/controls or outcomes	*Not serious (−0)*: No notable variations in population, intervention, or comparator were identified that were likely to have a meaningful impact on the outcomes	*Very serious (−2)*: The cumulative sample size across these studies was substantially less than *n* = 400	*Not suspected (−0)*: No indication of publication bias; the comprehensive search strategy successfully identified randomised controlled trials reporting both favourable and unfavourable results	*Very low* ⊕◯◯◯
Quality of life	Therapeutic exercise	5 (242)	RCT, pre‐post	*Very Serious (−2)*: These studies were considered to have a high risk of bias, due to outcome measurement (blinding) concerns	*Not serious (−0)*: No serious inconsistencies in findings were found across studies which could not be accounted for by differences in study populations, interventions, comparison/controls or outcomes	*Not serious (−0)*: No notable variations in population, intervention, or comparator were identified that were likely to have a meaningful impact on the outcomes	*Serious (−1)*: The cumulative sample size across these studies was somewhat less than *n* = 400	*Not suspected (−0)*: No indication of publication bias; the comprehensive search strategy successfully identified randomised controlled trials reporting both favourable and unfavourable results	*Very low* ⊕◯◯◯
Pain	Therapeutic exercise	8 (547)	RCT, pre‐post	*Very Serious (−2)*: These studies were considered to have a high risk of bias, due to outcome measurement (blinding) concerns	*Not serious (−0)*: No serious inconsistencies in findings were found across studies which could not be accounted for by differences in study populations, interventions, comparison/controls or outcomes	*Not serious (−0)*: No notable variations in population, intervention, or comparator were identified that were likely to have a meaningful impact on the outcomes	*Serious (−1)*: The cumulative sample size across these studies was greater less than *n* = 400, however some or all of the studies did not provide adequate indication of effect	*Not suspected (−0)*: No indication of publication bias; the comprehensive search strategy successfully identified randomised controlled trials reporting both favourable and unfavourable results	*Very low* ⊕◯◯◯
Jaw range of motion	Therapeutic exercise	3 (153)	RCT	*Very Serious (−2)*: These studies were rated high risk of bias, due to outcome measurement (blinding) concerns and/or reporting concerns	*Not serious (−0)*: No serious inconsistencies in findings were found across studies which could not be accounted for by differences in study populations, interventions, comparison/controls or outcomes	*Not serious (−0)*: No notable variations in population, intervention, or comparator were identified that were likely to have a meaningful impact on the outcomes	*Very serious (−2)*: The cumulative sample size across these studies was substantially less than *n* = 400	*Not suspected (−0)*: No indication of publication bias; the comprehensive search strategy successfully identified randomised controlled trials reporting both favourable and unfavourable results	*Very low* ⊕◯◯◯

## 4 Discussion

Therapeutic exercise is used widely as a treatment approach in TMD management. Previous systematic reviews have explored the effect of therapeutic exercise on clinical outcomes within the body functions and structures domain of the ICF (i.e., pain, range of motion, bite force) in individuals with TMD [33, 34, 35, 36, 37], however, none have investigated the effect of therapeutic exercise on wider domains of functioning such as activity, participation and further, QOL. The studies included in this systematic review encompassed a variety of exercise types and outcome measures. Overall, the evidence suggests that therapeutic exercise may improve activity, participation and QOL in people with TMD. Moreover, incorporating various exercise types, such as aerobic and jaw mobility exercises, appears to enhance these effects. The overall quality of evidence, as assessed using the GRADE framework, was very low. This underscores a need for caution in interpreting these findings and highlights the requirement for more rigorous studies to confirm these effects.

### 4.1 Interpretation of Findings

The current review evaluated the effectiveness of several different exercise programme delivery methods. Exercise interventions were delivered through home‐based programmes, supervised sessions, or a blend of both, and were often combined with other interventions including education, manual therapy and massage. Consistently, programmes where exercise was delivered alongside other interventions, such as education and manual therapy, demonstrated greater improvements than when delivered alone. This is consistent with current evidence for the management of musculoskeletal conditions [38]. With respect to the level of supervision, only one study compared the effectiveness of a home‐based exercise program to a combined supervised and home‐based program [31]. The results indicated that participants in the combined program experienced a greater reduction in patient‐reported activity limitations than those in the home‐based only group; however, neither group demonstrated significant change. These findings align with other musculoskeletal conditions including chronic neck pain [39] and lumbar spinal stenosis [40], where supervised therapeutic exercise has shown enhanced effectiveness compared to unsupervised programs. Häggman‐Henrikson and colleagues [41] have proposed that the improved outcomes from supervised exercise programs may stem from the emotional and social support provided, along with the ability to monitor patient adherence. This view is supported by recent clinical practice guidelines, which strongly recommend supervised exercise for managing chronic pain associated with TMD [6].

In this review, alongside varying delivery methods, a wide range of therapeutic exercise interventions were also examined. Some studies focused on localised, jaw‐specific exercises, while others assessed the benefits of more global exercises. Regarding jaw‐specific exercises, there was very low confidence in evidence that jaw mobility and jaw resistance exercises may improve activity, participation, QOL, pain and mouth opening ROM in individuals with TMD. This finding is consistent with recent literature regarding other musculoskeletal conditions such as neck pain [42]. While benefits were also observed across global types of exercise, such as aerobic, core stability and relaxation training, it is important to contextualise the positive effects of these more global exercise interventions in individuals with TMD. Core stability training was one aspect of a potentially effective exercise programme for TMD identified in this review; however, the addition of core stability training to jaw‐specific exercise and patient education did not lead to a greater improvement in QOL [29]. Conversely, aerobic training was found to amplify improvements in participation and QOL [26, 27]. The mechanisms through which exercise is proposed to enhance activity, participation and QOL are multifactorial, encompassing central and peripheral neurophysiological effects, social impacts and the enhancement of self‐efficacy [43]. This complex interplay of factors enhances an individual's capacity to engage in life situations, thereby improving QOL [43]. It also highlights the role that broader, global exercise interventions may play in the management of TMD.

### 4.2 Implications for Clinical Practice

Therapeutic exercise is strongly recommended in the management of TMD; however, to date, it has been unclear how exercise should be integrated and what effect exercise may have on wider domains of disability beyond pain and jaw ROM [6]. The findings of this review support the continued use of therapeutic exercise alone or in conjunction with other management approaches to improve activity, participation and QOL in patients with TMD.

Regarding specific types of therapeutic exercise, the findings of this review tentatively support the use of relaxation training, jaw mobility exercises and/or jaw resistance exercises to improve individuals' capacity to perform activities of daily life [22, 23, 28]. Relaxation training and aerobic exercise may be used to improve participation [27, 30]. Jaw mobility exercises with and without resistance may be used to improve QOL in those with TMD [20, 31]. Supervising the performance of exercise may enhance improvements to these outcomes [31].

It is essential to acknowledge that the evidence supporting these implications is considered very low. Consequently, the integration of these interventions in clinical settings should be considered within a wider framework of evidence‐based practice. Tailoring interventions as part of patient‐centered care is critical to ensure the feasibility of treatments and adherence to prescribed interventions [6]. When developing therapeutic exercise interventions for individuals with TMD, clinicians must consider patients' values and preferences [6]. Given the variability in TMD symptoms, clinicians must individually assess each patient's needs within the ICF framework to prescribe the most appropriate treatment. Such tailored approaches are critical, as they take into account the unique symptoms and activity and participation limitations of each patient, enhancing the potential for successful outcomes [44].

### 4.3 Strengths and Limitations

There are several strengths of this review which are important to consider. First, this review sourced articles from multiple databases, facilitating a thorough exploration of existing literature. Additionally, there was no restriction on date range, which allowed for a comprehensive collection of evidence. Two independent reviewers performed study screening, data extraction and quality assessment to ensure an in‐depth understanding of each study. Finally, studies were graded against robust and well‐established quality assessment tools to ensure validity of evidence.

A limitation was that certain databases did not allow for the application of prespecified limiters, leading to slight variation in search strategies across databases. The review also did not isolate TMD population subgroups; therefore, the application of these findings across known TMD subgroups remains unclear. It was beyond the scope of this review to compare the effect of different exercise interventions with each other and with other forms of treatment; thus, while exercise has been demonstrated to improve activity, participation and QOL measures, it remains unclear whether it is superior to other treatments. Finally, heterogeneity of interventions and outcomes across included studies limits the ability to form definitive conclusions on the degree of effect therapeutic exercise has on different domains of the ICF.

### 4.4 Implications for Future Research

This review found limited high‐quality evidence, suggesting a further need for well‐designed interventional studies examining the effect of therapeutic exercise on patient‐reported measures of activity, participation and QOL in individuals with TMD. Care should be taken to ensure the application of rigorous measures in the randomisation process, blinding where possible, ensuring high adherence, minimising drop out and reporting results according to the prespecified analysis plan. It is important to contextualise that all studies in this review were rated a high risk of bias due to the nature of the intervention and outcome measures used. As therapeutic exercise is typically prescribed by a health professional, it is difficult to maintain participant and intervention‐provider blinding to the intervention. Additionally, risk of bias is further increased when using patient‐reported outcomes as blinding of the outcome assessor is not possible. Future studies may consider using deception to withhold the true purpose of the study from participants.

As this is an emerging field of research, this review included a range of interventional study designs to provide a comprehensive sample of existing literature. As non‐randomised controlled trials and pre‐post studies were included, quality appraisal of certain outcomes began at ‘low’. As more evidence becomes available, future systematic reviews should consider limiting their search to only RCTs to provide the highest quality of evidence available [45].

While this review acknowledges that therapeutic exercise is often delivered within a multi‐modal package of care, future studies should consider including an isolated therapeutic exercise group to enable independent assessment of the intervention's within‐group effects. Future systematic reviews should also consider identifying the effect of therapeutic exercise interventions within certain populations of TMD. This may enhance the specificity of clinical guidelines for the management of TMD subgroups.

## 5 Conclusion

This systematic review has established that therapeutic exercise may be effective in improving activity, participation and QOL in individuals with TMD, although findings should be interpreted with caution due to very low certainty of evidence. A range of exercise types including local (mobility, resistance, relaxation) and global (aerobic, core stability) exercises was found to be beneficial.

## Author Contributions

**Sarah Scrase:** conceptualization, formal analysis, investigation, data curation, writing – original draft, writing – review and editing, visualisation. **Roma Forbes:** conceptualization, methodology, formal analysis, writing – review and editing, visualisation, supervision, project administration. **Adrienne Lindop:** formal analysis, investigation, data curation, writing – original draft, visualisation. **Adrienne Parcher:** formal analysis, investigation, data curation, writing – original draft, visualisation. **Louise Rainbird:** formal analysis, investigation, data curation, writing – original draft, visualisation. **Alana Dinsdale:** conceptualization, methodology, formal analysis, investigation, data curation, writing – review and editing, visualisation, supervision, project administration.

## Conflicts of Interest

The authors declare no conflicts of interest.

## Peer Review

The peer review history for this article is available at https://www.webofscience.com/api/gateway/wos/peer‐review/10.1111/joor.14042.

## Supporting information


Appendices S1–S4

## Data Availability

All other materials and supplementary documentation can be obtained by contacting the authors of this review.
