# Supplementary material for: Therapeutic Exercise Effects on Activity, Participation and Quality of Life in Individuals With Temporomandibular Disorders: A Systematic Review
Source: J Oral Rehabil. 2025 Jun 9;52(10):1839–64. doi: 10.1111/joor.14042 (PMC12426462; doi:10.1111/joor.14042)
Supplement: Supplementary file 1 — Appendices S1–S4 [file JOOR-52-1839-s001.docx]

APPENDICES

*Appendix 1: Search Strategy by Database*

Filters/Limiters = human study, peer reviewed & published in English. Pubmed, Embase, and Cochrane were able to limit to peer reviewed studies. Cochrane was also unable to limit to just human studies.

| **Database Name:**  PubMed  **Database Coverage:**  1946-Present  **Date of Last Search**:  10 April, 2024 | (“Temporomandibular Joint Dysfunction Syndrome”[MeSH Terms]) OR (“Temporomandibular Joint Disorders”[MeSH Terms]) OR (“Temporomandibular disorder*”[Title/Abstract]) OR (“Temporomandibular dysfunction”[Title/Abstract]) OR (“jaw pain”[Title/Abstract]) OR (“Temporomandibular pain”[Title/Abstract]) OR (“temporomandibular joint syndrome”[Title/Abstract])  AND  ("Exercise Therapy"[MeSH]) OR ("Exercise"[MeSH]) OR ("Exercise Movement Techniques"[MeSH]) OR (“Resistance Training"[MeSH Terms]) OR (“exercise”[Text Word]) OR (“resistance training”[Text Word]) OR (“movement therap*”[Text Word]) OR (“posture”[Text Word]) (“postural control”[Text Word]) OR (“postural training”[Text Word]) OR (“motor control” [Text Word]) OR (“active stretching”[Text Word]) OR (“flexibility”[Text Word]) OR (“relaxation training”[Text Word])  AND  (“Patient Participation”[MeSH]) OR (“Activities of Daily Living”[MeSH]) OR (“Pain”[MeSH]) OR (“Pain Measurement”[MeSH]) OR (“participation”[All Fields]) OR (“Oral Health-Related Quality of Life”[Text Word]) OR (“Jaw Functional Limitation Scale 8”[All Fields]) OR (“ADL”[Text Word]) OR (“activities of daily living”[Text Word]) OR (“activity of daily living”[Text Word]) OR (“participation restriction”[Text Word]) OR (“range of motion”[Text Word]) OR (“pain”[Text Word]) OR (“pain VAS”[Text Word]) OR (“mouth opening”[Text Word]) OR (“patient specific functional scale”[Text Word]) |
| --- | --- |
| **Database Name:**  CINAHL  **Database Coverage:**  1937-Present  **Date of Last Search**:  10 April, 2024 | (MH "Temporomandibular Joint Syndrome") OR (TI "temporomandibular joint disorder") OR (AB "temporomandibular joint disorder*") OR (TI “TMJ”) OR (AB “TMJ”) OR (TI “TMD”) OR (AB “TMD”) OR (TI “temporomandibular dysfunction”) OR (AB “temporomandibular dysfunction”) OR (TI “jaw pain”) OR (AB “jaw pain”) OR (TI “temporomandibular pain”) OR (AB “temporomandibular pain”) OR (TI “temporomandibular joint syndrome”) OR (AB “temporomandibular joint syndrome”)  AND  (MH "Therapeutic Exercise") OR (MH "Exercise") OR (MH "Resistance Training") OR (MH "Muscle Strengthening") OR (TX “movement therap*”) OR (TX exercise) OR (TX stretching) OR OR (TX “motor control”) OR (TX “postural control”) OR (TX “posture”) OR (TX “postural training”) OR (TX flexibility) OR (TX “relaxation training”) OR (TX “active stretching”)  AND  (MH "Social Participation") OR (MH "Activities of Daily Living") OR (MH “Range of Motion”) OR (MH “Pain”) OR (MH “Pain Assessment”) OR (TX “participation”) OR (TX "oral health related quality of life") OR (TX "jaw functional limitation scale 8") OR (TX "ADL*") OR (TX "activit* of daily living") OR (TX "activity limitation") OR (TX "participation restriction*”) OR (TX “movement”) OR (TX “pain”) OR (TX “VAS”) OR (TX “patient specific functional scale”) OR (TX “mouth opening”) |
| **Database Name:**  Embase  **Database Coverage:**  1947-Present  **Date of Last Search**:  10 April, 2024 | 'temporomandibular joint dysfunction syndrome'/de OR 'temporomandibular joint disorders'/de OR 'temporomandibular disorder*':ab,ti OR 'temporomandibular dysfunction':ab,ti OR 'jaw pain':ab,ti OR 'temporomandibular pain':ab,ti OR 'temporomandibular joint syndrome':ab,ti OR 'temporomandibular joint dysfunction syndrome'/exp OR 'temporomandibular joint disorders'/exp  AND  'kinesiotherapy'/de AND 'kinesiotherapy'/exp OR 'exercise':ab,ti OR 'resistance training':ab,ti OR 'movement therap*':ab,ti OR 'movement techniques':ab,ti OR 'posture':ab,ti OR 'motor control':ab,ti OR 'cervical exercis*':ab,ti OR 'active stretching':ab,ti OR 'flexibility':ab,ti OR 'relaxation training':ab,ti OR 'postural training':ab,ti  AND  'patient participation'/de OR 'range of motion'/de OR 'pain'/de OR 'pain assessment' OR 'daily life activity'/de OR 'activity of daily life assessment' OR 'participation':ab,ti OR 'oral health-related quality of life':ab,ti OR 'jaw functional limitation scale 8':ab,ti OR 'adl*':ab,ti OR 'activit* of daily living':ab,ti OR 'participation restriction':ab,ti OR 'range of motion':ab,ti OR 'range of movement':ab,ti OR 'movement':ab,ti OR 'pain':ab,ti OR ‘pain visual analog scale’:ab,ti OR 'vas':ab,ti OR 'patient specific functional scale':ab,ti OR 'psfs':ab,ti OR 'mouth opening':ab,ti |
| **Database Name:**  Cochrane  **Database Coverage:**  1996 - Present  **Date of Last Search**:  10 April, 2024 | ‘Temporomandibular joint disorders’[MeSH descriptor] OR (‘temporomandibular disorder’ OR ‘temporomandibular dysfunction’ OR ‘jaw pain’ OR ‘temporomandibular pain’ OR ‘temporomandibular joint’:ti,ab,kw)  AND  ‘Exercise’ [MeSH descriptor] OR ‘posture’ [MeSH descriptor] OR ‘resistance training’ [MeSH descriptor] OR ‘muscle stretching exercise’ [MeSH descriptor] OR (‘exercise’ ‘OR ‘resistance training’ OR ‘active stretching’ OR ‘flexibility’ OR ‘postural training’ OR ‘postural control’ OR ‘relaxation training’ OR ‘motor control’ OR ‘posture’:ti,ab,kw)  AND  ‘Patient participation’ [MeSH descriptor] OR ‘activities of daily living’ [MeSH descriptor] OR ‘pain’ [MeSH descriptor] OR ‘pain measurement’[MeSH descriptor] OR (‘participation’ OR ‘Oral Health-Related Quality of Life’ OR ‘Jaw Functional Limitation Scale 8’ OR ‘ADL’ OR ‘acitivit* of daily living’ OR ‘participation restriction’ OR ‘range of motion’ OR ‘pain’ OR ‘pain VAS’ OR ‘mouth opening’ OR ‘patient specific functional scale’:ti,ab,kw) |

*Appendix 2: Excluded Studies at full text stage of screening*

| **Number** | **Reference** | **Reason for Exclusion** |
| --- | --- | --- |
| 1 | Patil SR, Aileni KR. Effect of transcutaneous electrical nerve stimulation versus home exercise programme in management of temporomandibular joint disorder. *JCDR.* 2017;11(12):19-22. DOI: 10.7860/JCDR/2017/32761.10967 | Did not provide data associated with activity participation |
| 2 | Serna PD; Plaza-Manzano G, Cleland J, Fernández-de-las-Peñas C, Martin-Casas P, Diaz-Arribas MJ. Effects of Cervico-Mandibular Manual Therapy in Patients with Temporomandibular Pain Disorders and Associated Somatic Tinnitus: A Randomized Clinical Trial. *Pain Med.*  2020;21(3):613-624. DOI: 10.1093/pm/pnz278 | Intervention is multi-modal |
| 3 | Machado B, Mazzetto M, Da Silva M, de Falício C, Machado BCZ, Mazzetto MO, Da Silva MAMR, de Felício CM. Effects of oral motor exercises and laser therapy on chronic temporomandibular disorders: a randomized study with follow-up. *LIMS*. 2016;31(5):945-954. DOI: 10.1007/s10103-016-1935-6 | Intervention is multi-modal |
| 4 | Fricton J, Velly A, Ouyang W, Look JO. Does exercise therapy improve headache? A systematic review with meta-analysis. *Curr. Pain Headache Rep.* 2009;13(6):413-419. DOI: 10.1007/s11916-009-0081-2 | Not a randomised control trial or a pre-post trial |
| 5 | Da Costa LMR, Schimit EFD, Souza CWN, Edgar , de Souza da Silva L, Candotti CT, Loss JF. Effect of the Pilates method on women with temporomandibular disorders: A study protocol for a randomized controlled trial. *J Bodyw Mov Ther.* 2016;20(1):110-114. DOI: 10.1016/j.jbmt.2015.06.011 | Not a randomised control trial or a pre-post trial |
| 6 | Pehlivan TG, Yakut Y, Agirnas KE.  Investigation of effects different physiotherapy methods on pain and quality of life in patients with temporomandibular joint dysfunction. *Fiz. Rehabil.* 2018;29(2):S76 | Not written in English |
| 7 | Stechman-Neto J, Porporatti AL, Porto de Toledo I, Costa YM, Conti PCR, De Luca Canto G, Mezzomo LA. Stechman‐Neto, J. Porporatti, A. L. Porto de Toledo, I,  Costa, Y. M, Conti, P. C. R, De Luca Canto, G, Mezzomo, L. A. *J. Oral Rehabil.* 2016;43(6):468-479 DOI: 10.1111/joor.12380 | Not a randomised control trial or a pre-post trial |
| 8 | Yao L, Sadeghirad B, Li M, Li J, Wang Q, Crandon HN, Martin G, Morgain R, Florez ID, Hunskaar BS, Wells J, Moradi S, Zhu YJ, Ahmed MM, Gao Y, Cao L, Yang K, Tian J, Li J, Zhong L, Couban RJ, Gyatt GH, Agoritsas T, Busse JW. Management of chronic pain secondary to temporomandibular disorders: A systematic review and network meta-Analysis of randomised trials. *BMJ.* 2023(383). DOI: 10.1136/bmj-2023-076226 | Not a randomised control trial or a pre-post trial |
| 9 | Brantingham JW, Cassa TK, Bonnefin D, Pribicevic M, Robb A, Pollard H, Tong V, Korporaal C.Manipulative and multimodal therapy for upper extremity and temporomandibular disorders: A systematic review. *J. Manip. Physiol. Ther.* 2013;36(3):143-201. DOI: 10.1016/j.jmpt.2013.04.001 | Intervention is multi-modal |
| 10 | Ates K, Tonga E. Effect of cervical stabilization exercises on pain, mobility and functionality in patients with temporomandibular joint dysfunction. *Turk J Physiother Rehabil.* 2019;30(2):S24-S25. DOI: 10.1590/1678-775720150240 | Not a randomised control trial or a pre-post trial |
| 11 | Huhtela OS, Koivisto N, Hagg V, Sipila K. Effectiveness of applied relaxation method vs splint in treatment of temporomandibular disorders in Finnish students. *J. Oral Rehabil.* 2020;47(2):123‐131. DOI: 10.1111/joor.12884 | Did not provide data associated with activity participation |
| 12 | Nambi G, Abdelbasset WKB. Efficacy of Maitland joint mobilization technique on pain intensity, mouth opening, functional limitation, kinesiophobia, sleep quality and quality of life in temporomandibular joint dysfunction following bilateral cervicofacial burns. *Burns*. 2020;46(8):1880-1888. DOI: 10.1016/j.burns.2020.05.017 | Includes other medical conditions affecting the jaw |
| 13 | Bartley J. Breathing and temporomandibular joint disease. *J Bodyw Mov Ther.* 2011;15(3):291-297. DOI: 10.1016/j.jbmt.2010.06.002 | Not a randomised control trial or a pre-post trial |
| 14 | Grace EG, Sarlani E, Reid B, Grace EG, Sarlani E, Reid B. The use of an oral exercise device in the treatment of muscular TMD. *CRANIO.* 2002;20(3):204-208. DOI: 10.1080/08869634.2002.11746212 | Intervention is multi-modal |
| 15 | Kraaijenga S, van der Molen L, van Tinteren H, Hilgers F, Smeele L. Treatment of myogenic temporomandibular disorder: a prospective randomized clinical trial, comparing a mechanical stretching device (TheraBite®) with standard physical therapy exercise. *CRANIO.* 2014;32(3):208‐216. DOI: 10.1179/0886963413Z.00000000016 | Intervention is multi-modal |
| 16 | Häggman-Henrikson B, Wiesinger B, Wänman A. The effect of supervised exercise on localized TMD pain and TMD pain associated with generalized pain. *Acta Odontol. Scand.* 2018;76(1):6-12 DOI: 10.1080/00016357.2017.1373304 | Includes other medical conditions affecting the jaw |
| 17 | Moleirinho-Alves P, Benzinho T, Paco M. Effects of therapeutic exercise in TMDs with pain. *Ann. Med.* 2019;51:S225-S226. DOI: 10.1080/07853890.2018.1560739 | Not a randomised control trial or a pre-post trial |
| 18 | Lee IS, Kim SY. Effectiveness of manual therapy and cervical spine stretching exercises on pain and disability in myofascial temporomandibular disorders accompanied by headaches: a single-center cohort study. *BMC Sports Sci. Med. Rehabil.* 2023;15(1). DOI: 10.1186/s13102-023-00644-0 | Intervention is multi-modal |
| 19 | Zeno E, Griffin J, Boyd C, Oladehin, A, Kasser R. The effects of a home exercise program on pain and perceived dysfunction in a woman with TMD: a case study. *CRANIO.* 2001;19(4):279-288. | Not a randomised control trial or a pre-post trial |
| 20 | Lucas C, Branco I, Silva M, Alves P, Pereira AM. Benefits of manual therapy in temporomandibular joint dysfunction. *Ann. Med.* 2018;50:167-168. DOI: 10.1080/07853890.2018.1427445 | Not a randomised control trial or a pre-post trial |
| 21 | Atilgan E, Yildiz A, Kurt H, Algun C. Effect of yoga based exercises programme on pain, quality of life and depression with temporomandibular joint dysfunction. *Fiz. Rehabil.* 2015;26(2) | Not written in English |
| 22 | Brandao RAFS, Mendes CMC, Brandao Filho RA, De Sena EP. Isotonic exercises and relaxing techniques in individuals with temporomandibular dysfunction. *CRANIO*. 2022;40(3):199-206. DOI: 10.1080/08869634.2019.1708607 | Intervention is multi-modal |
| 23 | Storm Mienna C, Glas L, Magnusson M, Ilgunas A, Haggman-Herikson, Wanman A. Patients’ experiences of supervised jaw-neck exercise among patients with localised TMD pain or TMD pain associated with generalised pain. *Acta Odontol. Scand*. 2019;77(7):495-501. DOI: 10.1080/00016357.2019.1598573 | Includes other medical conditions affecting the jaw |
| 24 | Henriquez L, Plomer L, Leppe J, Tuttle N. Changes in symptoms and resting position of jaw, atlas and axis in patients with TMJ dysfunction following global postural reeducation. *Man. Ther*. 2016;25:103-104. DOI: 10.1016/j.math.2016.05.183 | Not a randomised control trial or a pre-post trial |
| 25 | Santos Miotto Amorim C, Firsoff EF, Vieira GF, Costa JR, Marques AP. Effectiveness of two physical therapy interventions, relative to dental treatment in individuals with bruxism: study protocol of a randomized clinical trial. *Trials*. 2014;15(1):8. DOI: 10.1186/1745-6215-15-8 | Not a randomised control trial or a pre-post trial |
| 26 | Wahlund K, Larsson Bp. Long-term treatment outcome for adolescents with temporomandibular pain. *Acta Odontol. Scand*. 2018;76(3):153-160. DOI: 10.1080/00016357.2017.1394490 | Intervention is multi-modal |
| 27 | Nicolakis P, Erdogmus B, Kopf A, Djaber-Ansari A, Piehslinger E, Fialka-Moser V. Exercise therapy for craniomandibular disorders. *Arch. Phys. M.* 200;81(9):1137-1142. DOI: 10.1053/apmr.2000.6282 | Intervention is multi-modal |
| 28 | Truelove E, Huggins KH, Mancl L, Dworkin SF. The efficacy of traditional, low-cost and nonsplint therapies for temporomandibular disorder: a randomised controlled trial. *JADA*. 2006;137(8):1099-1171. DOI: 10.14219/jada.archive.2006.0348. | Intervention is multi-modal |
| 29 | Xu L, Cai B, Lu S, Fan S, Dai K. The Impact of Education and Physical Therapy on Oral Behaviour in Patients with Temporomandibular Disorder: A preliminary Study. *Biomed Res. Int*. 2021:1-7. DOI: 10.1155/2021/6666680 | Intervention is multi-modal |
| 30 | Kanungo B, Patra RC, Mohanty P, Bawa P. Physical therapy approach in conjunction with dry needling on health related quality of life in patients with temporomandibular disorder: A randomised control trial. *Indian Journal of Public Health Research and Development*. 2020;11(7)187-192. https://doi.org/10.37506/ijphrd.v11i7.10077 | Intervention is multi-modal |
| 31 | Wahlund K, List T, Larsson B. Treatment of temporomandibular disorders among adolescents: a comparison between occlusal appliance, relaxation training, and brief information. *Acta Odontol Scand*. 2003;61(4):203-211. DOI: 10.1080/00016350310003891 | Did not provide data associated with activity participation |
| 32 | Henriquez L, Palomer L, Leppe J, Evans K. Global postural re-education and exercise therapy in the treatment of internal disorders of the temporomandibular joint. A case series. *Man. Ther.* 2016;25. DOI: 10.1016/j.math.2016.05.303 | Not a randomised control trial or a pre-post trial |
| 33 | Dickerson, SM, Weaver JM, Boyson AN, Thacker JA, Junak AA, Ritzline PD, Donaldson MB. The effectiveness of exercise therapy for temporomandibular dysfunction: a systematic review and meta-analysis. *Clin. Rehabil*. 2017;31(8):1039-1048. DOI: 10.1177/0269215516672275 | Not a randomised control trial or a pre-post trial |
| 34 | Simoes CASC, da Silva MAM, Magesty RA, Falci SGM, Douglas-de-Oliveira DW, Goncalves PF, Flecha OD. Counselling treatment versus counselling associated with jaw exercises in patients with disc displacement with reduction - a single-blinded, randomised, controlled clinical trial. *BMC Oral Health*. 2023;23(1):1-13. DOI: 10.1186/s12903-023-03096-7 | Did not provide data associated with activity participation |
| 35 | Moleirinho-Alves P, Almeida A, Cebola P, Oliveira R, Pezarat-Correia P. Effects of two programs with aerobic exercise in headache attributed to temporomandibular disorder. *J. Headache Pain*. 2021;22. DOI: 10.1186/s10194-021-01293-9 | Not a randomised control trial or a pre-post trial |
| 36 | Lindfors E, Magnusson T, Ernbery M. Patients’ experiences of therapeutic jaw exercises in the treatment of masticatory myofascial pain - A postal questionnaire study. J. Oral Rehabil*.* 2019;46(9):800-806. DOI: 10.1111/joor.12816 | Intervention is multi-modal |
| 37 | Carlson CR, Bertrand PM, Ehrlich AD, Maxwell AW, Burton RG. Physical self-regulation training for the management of temporomandibular disorders. *J. Orofac. Pain.* 2001;15(1):47-55 | Not a randomised control trial or a pre-post trial |
| 38 | Bakke M, Eriksson L, Thorsen NM, Sewerin I, Petersson A, Wagner A. Modified condylotomy versus conventional conservative treatment in painful reciprocal clicking – a preliminary prospective study in eight patients. *Clin. Oral Investig.* 2008;12(4):353-359. DOI: 10.1007/s00784-008-0204-x | Intervention is multi-modal |
| 39 | Sarfraz S, Anwar N, Tauqeer S, Asif T, Ul Ain N, Shakeel H. Comparison of effects of manual physical therapy and exercise therapy for patients with Temporomandibular disorders. *JPMA*.  2023;73(1):128-130. DOI: 10.47391/JPMA.3594 | Not a randomised control trial or a pre-post trial |
| 40 | Wig AD, Aaron LA, Turner JA, Huggins KH, Truelove E. Short-term clinical outcomes and patient compliance with temporomandibular disorder treatment recommendations. *J. Orofac. Pain.* 2004;18(3):203-213 | Not a randomised control trial or a pre-post trial |
| 41 | Baykan O, Narin S, Coskun Akar G. Comparative efficacy of kinesiotaping and stretching on sternocleidomastoid and upper trapezius muscles in patients suffering from myofascial pain due to temporomandibular joint disorder. *Apicare Journal.* 2022;26(5):695-701. DOI: 10.35975/apic.v26i4.2033 | Intervention is multi-modal |
| 42 | Kraaijenga S, van de Molen L, van Tinteren H, Hulgers F, Smeele L. Treatment of myogenic temporomandibular disorder: a prospective randomised clinical trial, comparing a mechanical stretching device (TheraBite(R)) with standard physical therapy exercise. *CRANIO*. 2014;32(3):208-216. DOI: 10.1179/0886963413Z.00000000016 | Intervention is multi-modal |
| 43 | Nicolakis P, Erdogmus B, Kopf A, Nicolakis M, Piehslinger E, Fialka-Moser V. Effectiveness of exercise therapy in patients with myofascial pain dysfunction syndrome. *J. Oral Rehabil.* 2002;29(4):362-368. DOI: 10.1046/j.1365-2842.2002.00859.x | Intervention is multi-modal |
| 44 | Navratil L, Navratil V, Hajkova S, Hlinakova P, Dostalova T, Vranova J. Comprehensive treatment of temporomandibular disorders. *CRANIO*. 2014;32(1):24-30. DOI: 10.1179/0886963413Z.0000000002 | Did not provide data associated with activity participation |

*Appendix 3: Risk of bias for pre-post studies using the NIH NHLBI Pre-Post Tool*

| Reference | 1.0 | 2.0 | 3.0 | 4.0 | 5.0 | 6.0 | 7.0 | 8.0 | 9.0 | 10.0 | 11.0 | 12.0 | Quality Rating |
| --- | --- | --- | --- | --- | --- | --- | --- | --- | --- | --- | --- | --- | --- |
| Kirschneck et al 2013^27^ | Yes | Yes | Cannot determine | Yes | Not reported | Cannot determine | Yes | Not reported | Yes | Yes | No | Not reported | Poor |
| Moleirinho-Alves et al 2021^21^ | Yes | Yes | No | Yes | Yes | Cannot determine | Yes | No | No | Yes | Yes | Not applicable | Poor |

*Appendix 4: Risk of bias for non-randomised controlled trials using the ROBINS-I Tool*

| **Reference** | **D1** | **D2** | **D3** | **D4** | **D5** | **D6** | **D7** | **Overall** |
| --- | --- | --- | --- | --- | --- | --- | --- | --- |
| Moleirinho-Alves et al 2021^29^ | 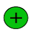 | 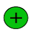 | 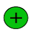 | 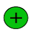 | 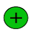 | 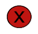 | 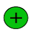 | 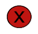 |


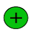
 *= low risk of bias*

*
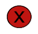
= serious risk of bias*
